# Regulatory roles of interferon-inducible protein 204 on differentiation and vasculogenic activity of endothelial progenitor cells

**DOI:** 10.1186/s13287-016-0365-5

**Published:** 2016-08-11

**Authors:** Junjie Yang, Xiaofei Zhang, Zhenao Zhao, Xizhe Li, Xu Wang, Ming Chen, Bo Song, Masaaki Ii, Zhenya Shen

**Affiliations:** 1 Institute for Cardiovascular Science & Department of Cardiovascular Surgery of The First Affiliated Hospital, Soochow University, 188 Shizi Street, Suzhou, 215006 China; 2 Department of Cardiovascular Surgery, Affiliated Shanghai 1st People’s Hospital, Shanghai Jiaotong University, Shanghai, 200080 China; 3 Suzhou Key Laboratory of Macromolecular Design and Precision Synthesis, Jiangsu Key Laboratory of Advanced Functional Polymer Design and Application, College of Chemistry, Chemical Engineering and Materials Science, Soochow University, Suzhou, 215006 China; 4 Division of Research Animal Laboratory and Translational Medicine, Research and Development Center, Osaka Medical College, 2-7 Daigaku-machi, Takatsuki, Osaka 569-8686 Japan; 5 Institute for Cardiovascular Science, Soochow University, 708 Renmin Road, Suzhou, 215006 China

**Keywords:** Endothelial progenitor cells, Ifi204, Endothelial differentiation, Vasculogenesis, Hindlimb ischemia

## Abstract

**Background:**

Endothelial progenitor cells (EPCs) have shown great potential in angiogenesis either by their differentiation into endothelial cells or by secretion of angiogenic factors. Interferon-inducible protein 204 (Ifi204) has been reported to participate in the regulation of cell growth and differentiation. However, its role in differentiation of EPCs remains unknown. We proposed that Ifi204 could modulate the differentiation and regenerative abilities of EPCs.

**Methods:**

Ifi204-expressing lentivirus and Ifi204 siRNA were introduced into EPCs to overexpress and suppress the expression of Ifi204. Using fluorescence-activated cell sorting, immunocytochemistry, and quantitative PCR, endothelial markers including CD31, VE-cadherin, and vWF were detected in the modified EPCs. An in-vitro incorporation assay and a colony-forming assay were also performed.

**Results:**

Evidence showed that Ifi204 inhibition decreased the endothelial differentiation and vasculogenic activities of EPCs in vitro. In mice with hindlimb ischemia, downregulation of Ifi204 in EPCs, which was tracked by our newly synthesized nanofluorogen, impaired neovascularization, with a corresponding reduction in hindlimb blood reperfusion by postoperative day 14.

**Conclusions:**

Ifi204 is required for EPC differentiation and neovascularization in vitro and in vivo. The regulatory roles of Ifi204 in EPC differentiation may benefit the clinical therapy of ischemic vascular diseases.

**Electronic supplementary material:**

The online version of this article (doi:10.1186/s13287-016-0365-5) contains supplementary material, which is available to authorized users.

## Background

In 1997, Asahara et al. [1] revolutionized the traditional concept of postnatal neovascularization, proposing that immature endothelial progenitor cells (EPCs) circulate in the adult blood and participate in postnatal angiogenesis. This idea was met with great enthusiasm because it suggested that EPCs could be derived from peripheral blood and used as a novel therapy in patients with vascular disease, and thus was followed by booming research on EPCs over the last decades. Upon ischemic stimuli, EPCs are recruited to ischemic tissue and enhance angiogenesis either by incorporation into neovascularization [2–5] or by secreting angiogenic and cytoprotective cytokines and growth factors [6–10]. To optimize therapeutic outcomes, researchers have developed techniques such as genetic modification of EPCs and so on [11–14].

Interferon-inducible protein 204 (Ifi204), a member of the interferon-inducible p200 family of proteins, has participated in the regulation of cell growth and differentiation [15]. Evidence has shown that Ifi204 could promote muscle cell differentiation by sequestering regulatory factors that block the differentiation process [16, 17]. Very recently, our group reported that Ifi204 is downregulated in the bone marrow cells of diabetes mellitus type 2 mice [18]. Because EPCs in diabetes mellitus have been reported to be impaired in regenerative potency, we thus proposed that Ifi204 could modulate the differentiation and regenerative abilities of EPCs. Through overexpression and inhibition of Ifi204 in EPCs, we examined the role of Ifi204 in EPC differentiation and vasculogenesis ability in vitro, and their ability for neovascularization in a hindlimb ischemia model.

## Methods

### Animals

All animal procedures were approved by the Ethic Committee of Soochow University and carried out in accordance with the Guidelines for the Care and Use of Research Animals established by Soochow University. C57/BL6 mice (8 weeks old, male) were housed at the Animal Facility of Soochow University on a 12-hour light/dark cycle with free access to water and standard mouse food.

### Isolation and identification of EPCs

Bone marrow cells were isolated from mouse bones as described previously [19]. Briefly, cells were collected from all bones smashed with a mortar and a pestle, and were further isolated by density-gradient centrifugation with Histopaque 1083 (Sigma, St. Louis, MO, USA). Cells were cultured in Endothelial Cell Basal Medium-2 (Lonza, Basel, Switzerland), supplemented with EGM-2 MV SingleQuots (Lonza) and 10 % fetal bovine serum. All the floating cells were washed out with fresh culture medium 2 days after cell plating, and were further cultured for 14 days before experiments. Then 5 μg/ml of DiI-Ac-LDL solution (Biomedical Technologies Inc., Stoughton, MA, USA) was added to the cells and culture continued for 4 hours. The cells were then fixed by 4 % paraformaldehyde (PFA)/PBS and stained with DAPI (Beyotime Biotechnology, Shanghai, China). Endocytosis of Ac-LDL was observed under a fluorescent microscope (Olympus, Tokyo, Japan).

### Transfection of Ifi204 in EPCs

Mouse Ifi204 siRNAs were synthesized by GenePharma Co. Ltd (Shanghai, China). The sequences for Ifi204 siRNAs were designed as follows: sense (5′ to 3′), CCAACAAUGGUUAUCUCAATT; and anti-sense (5′ to 3′), UUGAGAUAACCAUUGUUGGTT. Cell transfection was performed with Lipofectamine 2000 (Invitrogen, Carlsbad, CA, USA), according to the manufacturer’s protocols. Briefly, the cells in 60 mm dishes were transfected with 200 pmol of Ifi204 siRNA and the scramble siRNA separately. Forty-eight hours post transfection, the cells were harvested and the efficiency of transfection was confirmed using reverse transcription quantitative PCR (RT-qPCR). At the same time, the transfected cells were further used in the in-vitro assays.

### Synthesis of lentivirus overexpressing Ifi204 and transduction of lentivirus in EPCs

Ifi204 full-length CDS was cloned into the *Not*I/*Bam*HI sites of the LV5 vector (EF1A-GFP-PURO). Positive clones of LV5-Ifi204 vectors were identified by DNA sequencing. Recombinant LV5 lentivirus containing Ifi204 gene was produced in the HEK 293 packaging cell line. For infection, cells were exposed to viral supernatant at a multiplicity of infection (MOI) of 50 for 24 hours. The efficiency of transduction was estimated by calculating the percentage of green fluorescence protein (GFP)-positive cells and RT-qPCR.

### Fluorescence-activated cell sorting analysis

To detect endothelial lineage antigens on EPCs, fluorescence-activated cell sorting (FACS) analysis was conducted 4 days after siRNA transfection or lentivirus transduction of EPCs. Surface expression of CD133 and VE-cadherin was determined by phycoerythrin (PE)-conjugated antibody (e-Bioscience, San Diego, CA, USA) against mouse CD133 and VE-cadherin. FITC-conjugated anti-mouse PECAM (CD31) antibody (e-Bioscience) was used to determine the surface expression of CD31. Isotype-identical antibodies served as negative controls (BioLegend, San Diego, CA, USA). The staining was performed for 20 minutes at 4 °C, followed by quantitative analysis with MoFlo XDP (Beckman Coulter, Brea, CA, USA).

### Immunocytochemistry

Attached cells were briefly fixed with ice-cold acetone and blocked in antibody dilution buffer (2 % BSA/PBS) for 1 hour at room temperature. After removal of the blocking solution, primary antibodies anti-VE-cadherin antibody (1:100; Santa Cruz, Dallas, TX, USA) and anti-CD31 antibody (1:100; Santa Cruz) were added and cells were incubated at 4 °C overnight. The cells were then washed and incubated with Alexa 488 donkey anti-goat IgG and Alexa 488 goat anti rabbit IgG (Invitrogen, Carlsbad, CA, USA) for 30 minutes at room temperature. Immunofluorescence was observed under a fluorescent microscope (Olympus).

### Real-time RT-PCR analysis

Cellular mRNA was extracted from each sample using PureLink RNA Mini Kit (Ambion, Carlsbad, CA, USA) according to the manufacturer’s instructions. cDNA was synthesized using PrimeScript RT reagent Kit (TAKARA, Tokyo, Japan). For qPCR, the converted cDNA samples (2 μl) were amplified in a final volume of 10 μl using SYBR Green Master Mix reagent (Applied Biosystems, Foster City, CA, USA) and gene-specific primers with the StepOne Plus real-time PCR system (Applied Biosystems). Sequences of the specific primers used in RT-PCR are presented in Table 1. The mean cycle threshold values were used to calculate gene expression levels with normalization to mouse GAPDH. Quantitative analyses were based on three independent biological samples.

**Table 1:** Primer sequences for real-time quantitative PCR

Gene name	Forward primer	Reverse primer
*GAPDH*	ACATCATCCCTGCATCCACT	CACATTGGGGGTAGGAACAC
*CD31*	TGGCCAGTCACTTGAAGACA	CAGTTGTTGTAGCCAGCCATT
*VE-cadherin*	TACTCAGCCCTGCTCTGGTT	GCTTGCAGAGGCTGTGTCTT
*vWF*	ACGCCATCTCCAGATTCAAG	AAGCATCTCCCACAGCATTC
*VEGF*	AGCACAGCAGATGTGAATGC	AATGCTTTCTCCGCTCTGAA

### In-vitro vasculogenesis assay

Human umbilical cord-derived endothelial cells (HUVECs) and EPCs with Ifi204 siRNA transfection or LV5-Ifi204 infection were used for tube formation assays as described previously [19–21]. Fifty microliters of chilled Matrigel matrix (BD, Bedford, MA, USA) was added into each well of the 96-well plate and incubated at 37 °C for 30 minutes. EPCs were labeled with DiI (Invitrogen, Eugene, OR, USA) for 20 minutes. HUVECs together with DiI-labeled EPCs in 100 μl of 10 % FBS/EGM-2MV medium were added to each well. The number of incorporated DiI-labeled cells into tubes was counted under a fluorescent microscope (Olympus).

### EPC colony-formation assay

EPCs with inhibition or overexpression of Ifi204 were cultured in methylcellulose-based medium (MethoCultM3236; Stem Cell Technologies, Vancouver, BC, Canada) supplemented with VEGF (50 ng/ml; PeproTech, Rocky Hill, NJ, USA), SCF (100 ng/ml; PeproTech), IL-3 (20 ng/ml; PeproTech), EGF (50 ng/ml; PeproTech), bFGF (50 ng/ml; PeproTech), IGF-1 (50 ng/ml; PeproTech), and 30 % FBS (Sigma). EPC colonies were manually counted under a phase contrast microscope (Olympus) after culturing 1000 cells for 14 days. Endothelial phenotype of the EPC colonies was confirmed by uptake of DiI-Ac-LDL and FITC-conjugated isolectin B4 (Sigma Chemical Co., Milwaukee, WI, USA).

### Hindlimb ischemia model and cell transplantation

Mice aged 8–10 weeks were anesthetized with 160 mg/kg pentobarbital i.p. The common femoral artery, including the superficial and other arterial branches as well as veins, were ligated and excised to generate the hindlimb ischemia model as described previously [22]. EPCs were administered intramuscularly immediately after induction of ischemia. For the study of EPC tracking, cells were incubated with our newly synthesized nanofluorogen [23], a bolaamphiphile with a tetraphenylethene (TPE) unit attached and two pyridinium salt terminated alkyl groups (TPE-11). Cells were washed with PBS and incubated with TPE-11 at a concentration of 16 μg/ml overnight. The cells were then collected and 50,000 cells were injected into the gastrocnemius muscle (two sites) and lower ligation sites (two sites) per mouse. Laser Doppler perfusion imaging (LDPI; Perimed Instruments AB, Stockholm, Sweden) was conducted to measure blood flow recovery ratio (ischemia/nonischemia). The perfusions of the ischemic and nonischemic limbs were calculated on the basis of colored histogram pixels, and expressed as the ratio of ischemic to nonischemic hindlimb perfusion.

### Detection of neovascularization in the hindlimb ischemia model

To visualize functional vessels with blood flow, Griffonia (Bandeiraea) Simplicifolia Lectin I (BSL I; Vector Laboratories, Burlingame, CA, USA) was infused into mouse heart 15 minutes before sacrifice. Ischemic muscle samples were embedded in OCT compound (Sakura, Tokyo, Japan), snap-frozen in liquid nitrogen, and cut at a thickness of 6 μm with a Leica CM 1950 Cryomicrotome (Carl Zeiss AG, Jena, Germany). Frozen sections were incubated with anti-BSL I antibody (Vector Laboratories) overnight at 4 °C, followed by staining with Alexa fluor 594-conjugated anti-rabbit antibody (Invitrogen) to identify capillaries in the ischemic tissue. Images were examined with a fluorescent microscope (Olympus). Cell incorporation into neovascularization was detected by double-positivity of BSL I and TPE-11.

### Statistical analysis

Comparison of multiple groups was performed by ANOVA. Two-group analysis was performed by Student *t* test. Values were expressed as means ± SEM. *P* < 0.05 was considered significant. Continuous variables are expressed as mean ± SD or SEM.

## Results

### EPC culture and regulation of Ifi204 expression in EPCs

**Fig. 1:**
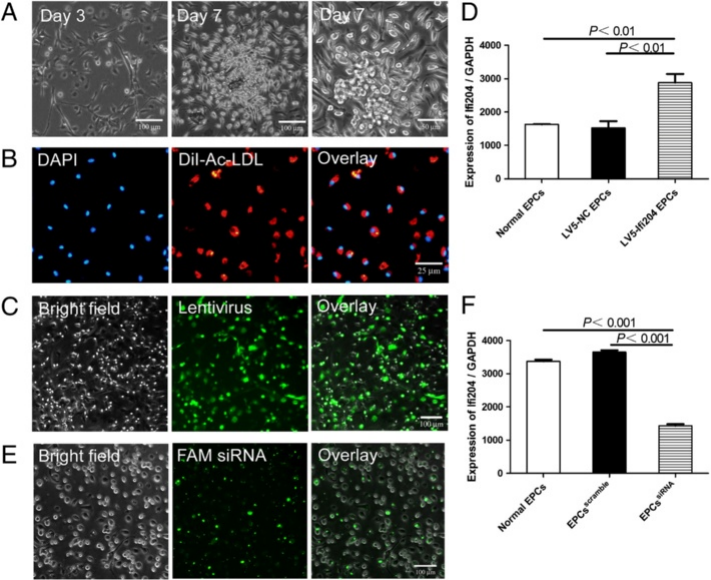
Culture of EPCs and modulation of Ifi204 in EPCs. **a** Morphology of mouse bone marrow-derived EPCs cultured at day 3 and day 7. *Bar*, 100 μm and 50 μm. **b** Uptake of DiI-Ac-LDL by EPCs. *Bar*, 25 μm. **c** Infection of Ifi204 lentivirus in EPCs. *Bar*, 50 μm. **d** Expression of Ifi204 in EPCs after transduction of Ifi204 expressing lentivirus (*n* = 3). *P* < 0.01, LV5-Ifi204 EPCs vs LV-NC EPCs and normal EPCs. **e** Transfection of Ifi204 siRNA in EPCs. *Bar*, 100 μm. **f** Expression of Ifi204 in EPCs after transfection of Ifi204 siRNA (*n* = 3). *P* < 0.001, EPCs^siRNA^ vs normal EPCs and EPCs^scramble^. *EPC* endothelial progenitor cell

In the culture medium of 10 % FBS/EGM-2MV, adherent cells changed their morphology to spindle shape after 3 days. Seven days later, most of the cells formed colonies as reported previously (Fig. 1a). As a characteristic of EPCs, cells could uptake Dil-Ac-LDL (Fig. 1b). We constructed a lentivirus vector with Ifi204 full-length CDS cloned into the *Not*I/*Bam*HI sites. EPCs were infected with LV5-Ifi204 for 24 hours (Fig. 1c). The expression of Ifi204 in EPCs after lentivirus transduction reached about twofold compared with that of normal EPCs (Fig. 1d). We also transfected Ifi204 siRNA into EPCs (Fig. 1e), which inhibited the expression of Ifi204 by 58 ± 0.03 % (Fig. 1f).

### Downregulation of Ifi204 inhibited EPC differentiation

**Fig. 2:**
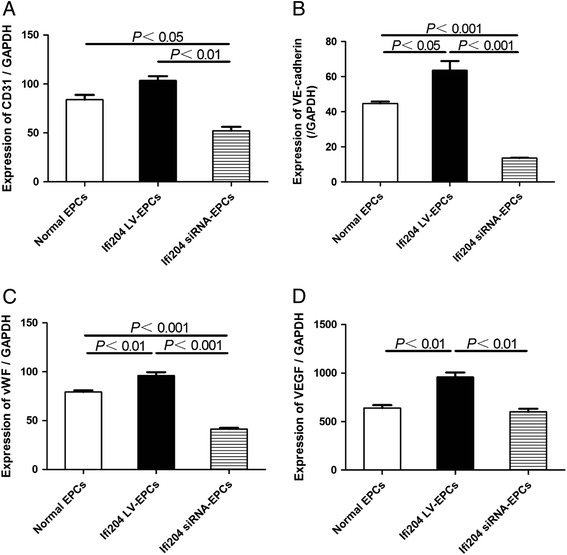
qPCR analysis of endothelial markers and growth factors in different EPC groups. mRNA expression levels of CD31 **a**, VE-cadherin **b**, vWF **c**, and VEGF **d** in 14-day cultured cells were assessed by quantitative real-time RT-PCR. The mRNA expressions were normalized to GAPDH (*n* = 3). All assays were performed in triplicate and demonstrated similar results. *EPC* endothelial progenitor cell

**Fig. 3:**
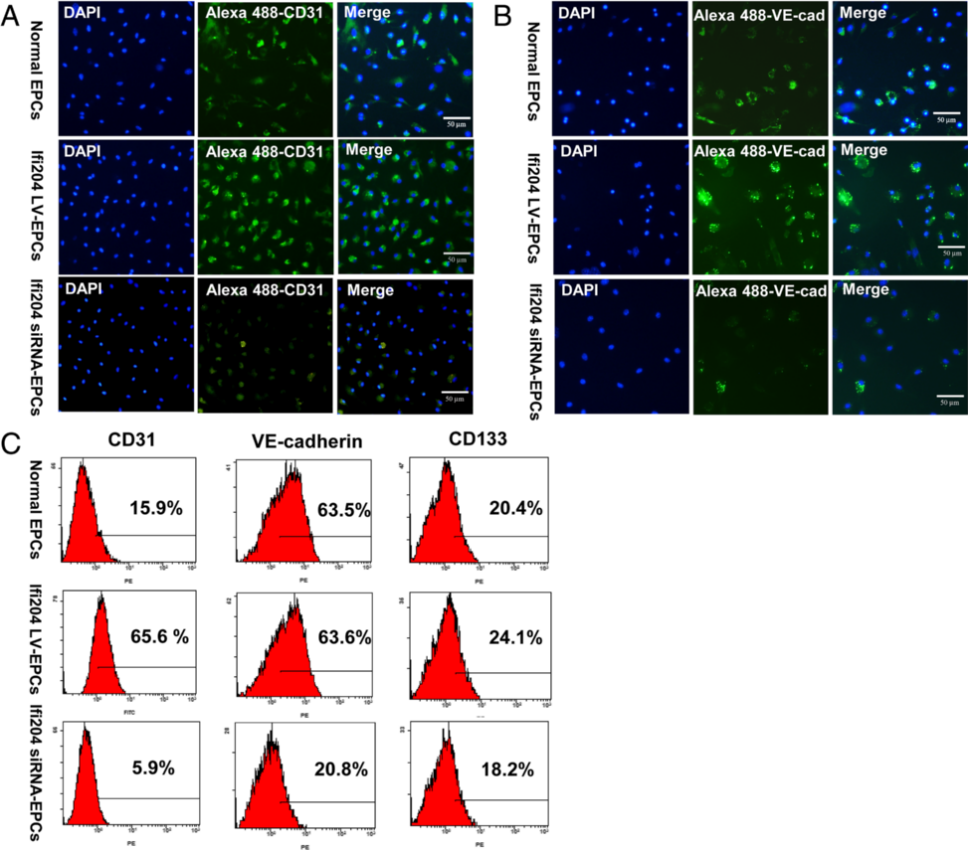
Assessment of endothelial markers in cultured EPCs by immunocytostaining and FACS. Cells were cultured and induced with Ifi204 lentivirus or Ifi204 siRNA. Adherent cells were stained with endothelial marker CD31 **a** and VE-cadherin **b**. *Bar*, 50 μm. **c** Cell surface markers investigated by FACS were as follows: endothelial cell (CD31 and VE-cadherin) and stem cell (CD133). Cellular surface markers were analyzed on normal EPCs (*upper panel*), Ifi204 lentivirus-transduced EPCs (*middle panel*), and Ifi204 siRNA-transfected EPCs (*lower panel*). Percentages of each marker indicated in the histograms (*n* = 3). *EPC* endothelial progenitor cell

We next determined the expression profiles of endothelial cell-lineage markers in modified EPCs in comparison with normal EPCs. As shown in Fig. 2, the mRNA expression levels of CD31, VE-cadherin, vWF, and VEGF in Ifi204 LV-transduced EPCs were at least twofold those in Ifi204 siRNA-transfected EPCs (CD31 and VEGF in Ifi204 LV-EPCs vs Ifi204 siRNA-EPCs, *P* < 0.01; VE-cadherin and vWF in Ifi204 LV-EPCs vs Ifi204 siRNA-EPCs, *P* < 0.001), and also higher than those in normal EPCs (vWF and VEGF in Ifi204 LV-EPCs vs normal EPCs, *P* < 0.01; VE-cadherin in Ifi204 LV-EPCs vs normal EPCs, *P* < 0.05). Most of the Ifi204 LV-transduced EPCs expressed CD31 (Fig. 3a) and VE-cadherin (Fig. 3b), but they were dramatically decreased in Ifi204 siRNA-transfected EPCs. We also analyzed the expression of endothelial cell-lineage markers on EPCs by FACS (Fig. 3c). EPCs infected with Ifi204 lentivirus had higher expression of CD31, VE-cadherin, and CD133 than normal EPCs. In contrast, inhibition of Ifi204 reduced the expression of these cell surface markers in EPCs. These results demonstrated a differentiation inhibitory effect of Ifi204 knockdown in EPCs.

### In-vitro vasculogenic activity of EPCs was modulated by the expression of Ifi204

**Fig. 4:**
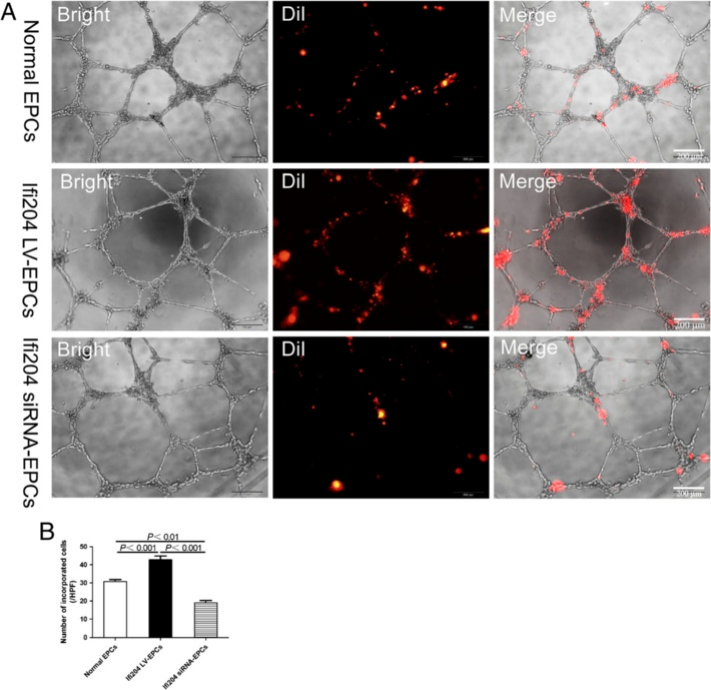
Tube formation assay by HUVECs incorporated with modified EPCs. DiI-labeled cells and HUVECs were seeded onto Matrigel-coated 96-well plates in 10 % FBS/EBM2-MV without growth factors. After 24 hours in culture, incorporation of each cell population into tube-like structures formed with HUVECs was evaluated under fluorescence microscopy. **a** Morphology of cultured HUVECs forming a network on Matrigel with the incorporation of DiI-positive cells. *Bar*, 200 μm. **b** Number of incorporated DiI-positive cells into tubular structures was counted and averaged. All assays demonstrated similar results (*n* = 4). *EPC* endothelial progenitor cell

To determine whether Ifi204 could modulate the vasculogenic activity of EPCs, we carried out a tube-forming assay on Matrigel using HUVECs and EPCs as described previously [19–21]. As shown in Fig. 4, overexpression of Ifi204 in EPCs significantly enhanced the numbers of incorporated cells into tube structures of HUVECs (*P* < 0.001, Ifi204 LV-EPCs: 43 ± 4 cells/HPF vs normal EPCs: 31 ± 2 cells/HPF and Ifi204 siRNA-EPCs: 19 ± 3 cells/HPF). Inhibition of Ifi204 in EPCs, however, prohibited the incorporation of EPCs into tube structures of HUVECs. The in-vitro angiotube assay revealed that the expression level of Ifi204 in EPCs was closely correlated with the angiogenesis abilities of the cells.

### Colony-forming ability of EPCs was slightly enhanced by Ifi204 siRNA transfection

**Fig. 5:**
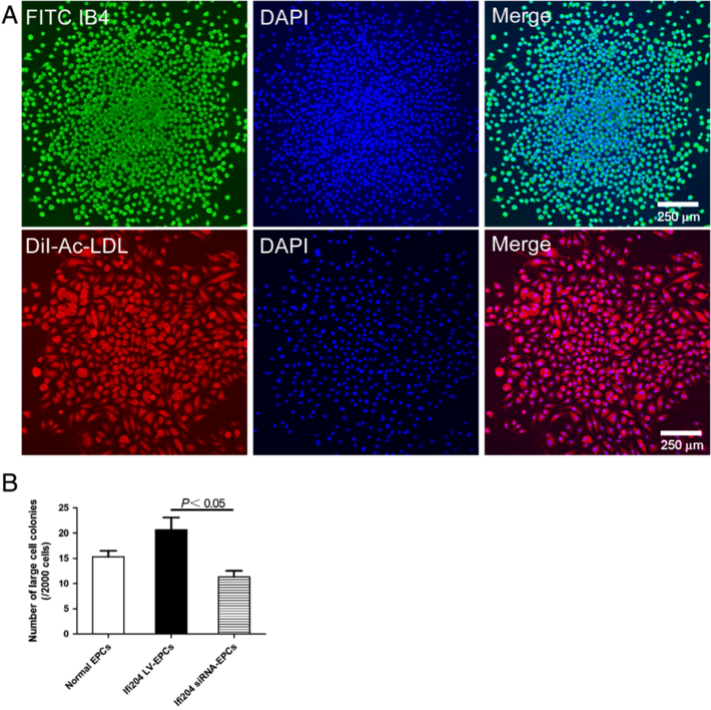
Colony-forming assay by different EPC groups. **a** Representative staining for fluorescein isolectin B4 (ILB4) and DiI-Ac-LDL in EPC colonies. *Bar*, 250 μm. **b** Large cell colonies were counted and analyzed in each group. All assays demonstrated similar results (*n* = 4). *EPC* endothelial progenitor cell

Subsequently, we explored the influence of Ifi204 on colony-formation ability, representing functional stem cell parameters. Colonies formed by EPCs could uptake FITC isolectin B4 and DiI-Ac-LDL (Fig. 5a). In EPCs, overexpression of Ifi204 increased the numbers of colonies by about twofold compared with downexpression of Ifi204 (*P* < 0.05, Ifi204 LV-EPCs: 21 ± 4 vs Ifi204 siRNA-EPCs: 11 ± 2), without reaching a significant difference when compared with nonmodified EPCs (Fig. 5b).

### Reperfusion of blood flow in ischemic hindlimb was hindered by transplantation of Ifi204 siRNA-transfected EPCs

**Fig. 6:**
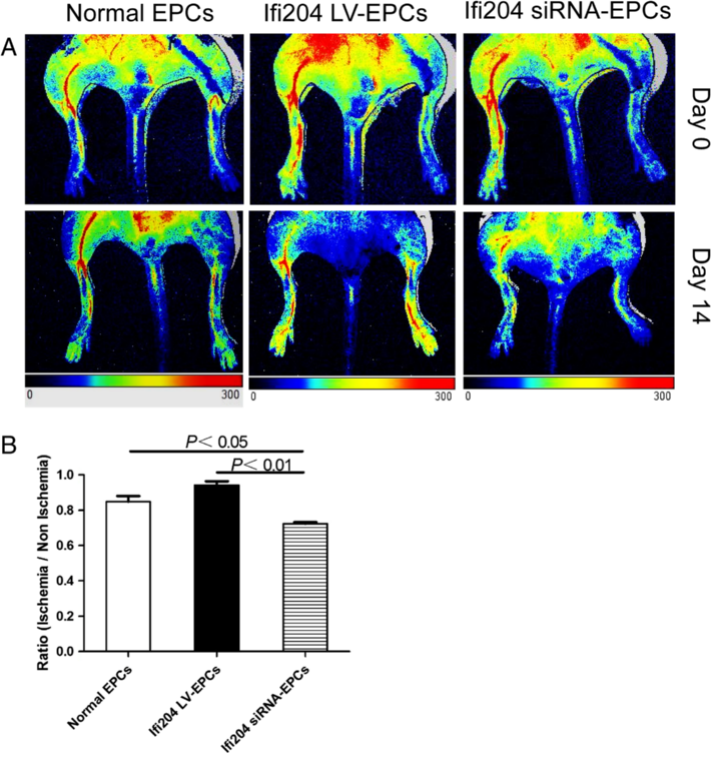
Blood flow patterns in ischemic hindlimbs. **a** LDPI was used to analyze blood flow 14 days after ischemia. *Top panel*: representative features just after surgery. Colors displayed correspond to intervals of perfusion value from 0 (*dark*) to 300 (*red*). *Red*, highest velocity; *green*, intermediate; *blue*, low; and *dark*, lowest velocity. **b** Blood-flow ratio of ischemic-to-contralateral hindlimb after 14 days (*n* = 6). *P* < 0.05, normal EPCs vs Ifi204 siRNA-EPCs; *P* < 0.01, Ifi204 LV-EPCs vs Ifi204 siRNA EPCs. *EPC* endothelial progenitor cell (Color figure online)

To evaluate the neovascularization in response to Ifi204 modulation in EPCs, we used the hindlimb ischemia model. The laser Doppler blood flow measurement was performed immediately after ligation and 14 days after surgery and cell transplantation (Fig. 6a). The blood flow recovery ratios of ischemia to nonischemia were used as an index of the neovascularization following hindlimb ischemia. Initial reductions in perfusion were similar in all groups (Fig. 6a). Perfusion recovered incrementally over a 2-week period. Transplantation with normal EPCs and Ifi204 LV-EPCs presented similar blood flow recovery (normal EPCs: 85 ± 0.05 % vs. Ifi204 LV-EPCs: 93 ± 0.03 %, NS) (Fig. 6a, b). In comparison with this, transplantation with Ifi204 siRNA-transfected EPCs had significantly less blood flow recovery as assessed by laser Doppler (*P* < 0.05, Ifi204 siRNA-EPCs: 72 ± 0.02 % vs normal EPCs: 85 ± 0.05 %; *P* < 0.01, Ifi204 siRNA-EPCs: 72 ± 0.02 % vs Ifi204 LV-EPCs: 93 ± 0.03 %), indicating that interference of Ifi204 expression in EPCs may prohibit their vascular reparative ability.

### In-vivo vasculogenic activity of EPCs was modulated by the expression of Ifi204

**Fig. 7:**
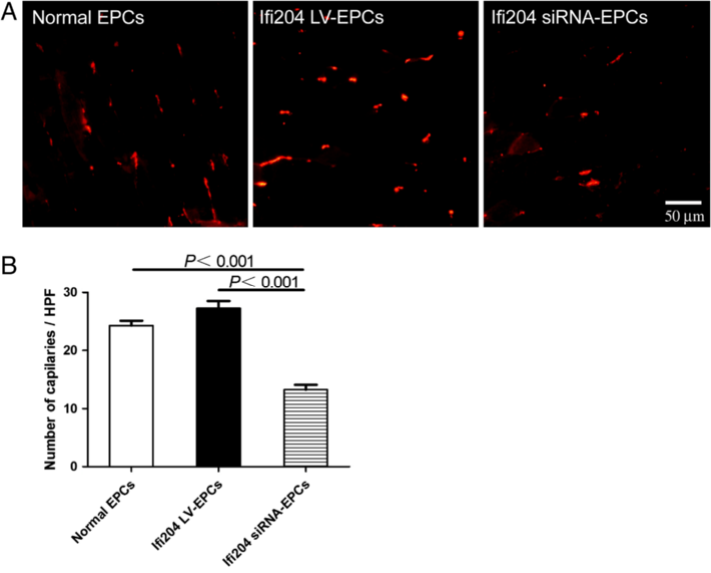
Histological analysis for capillary density in ischemic hindlimb. Cells were intramuscularly injected immediately after hindlimb ischemia. Muscle samples were harvested following BSL I systemic perfusion 14 days after surgery. **a** Representative images of vessel morphology from each treatment group. *Bar*, 50 μm. **b** Numbers of capillaries were counted in the ischemic area and averaged in each group (*n* = 4), respectively. *EPC* endothelial progenitor cell

**Fig. 8:**
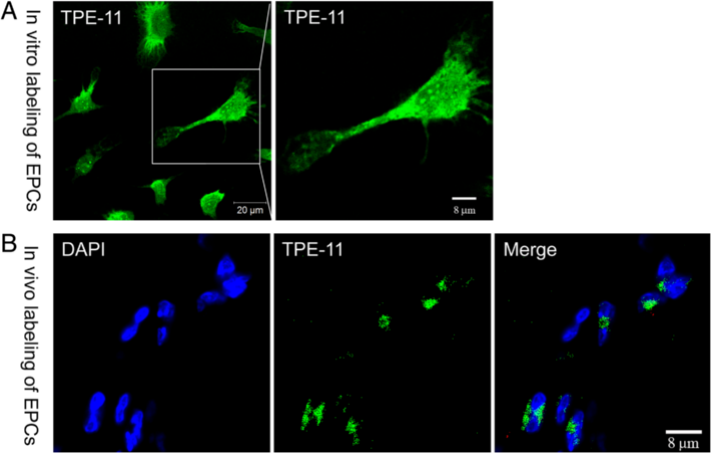
In-vitro and in-vivo staining of EPCs by the nanofluorogen. **a** EPCs were incubated with TPE-11 for 12 hours. Fluorescent images of the cells observed by a confocal microscope at the 405 nm excitations. *Bar*, 20 μm and 8 μm. **b** Sections of the ischemic gastrocnemius muscle were observed by a confocal microscope at the 405 nm excitations. *Bar*, 8 μm. *EPC* endothelial progenitor cell

**Fig. 9:**
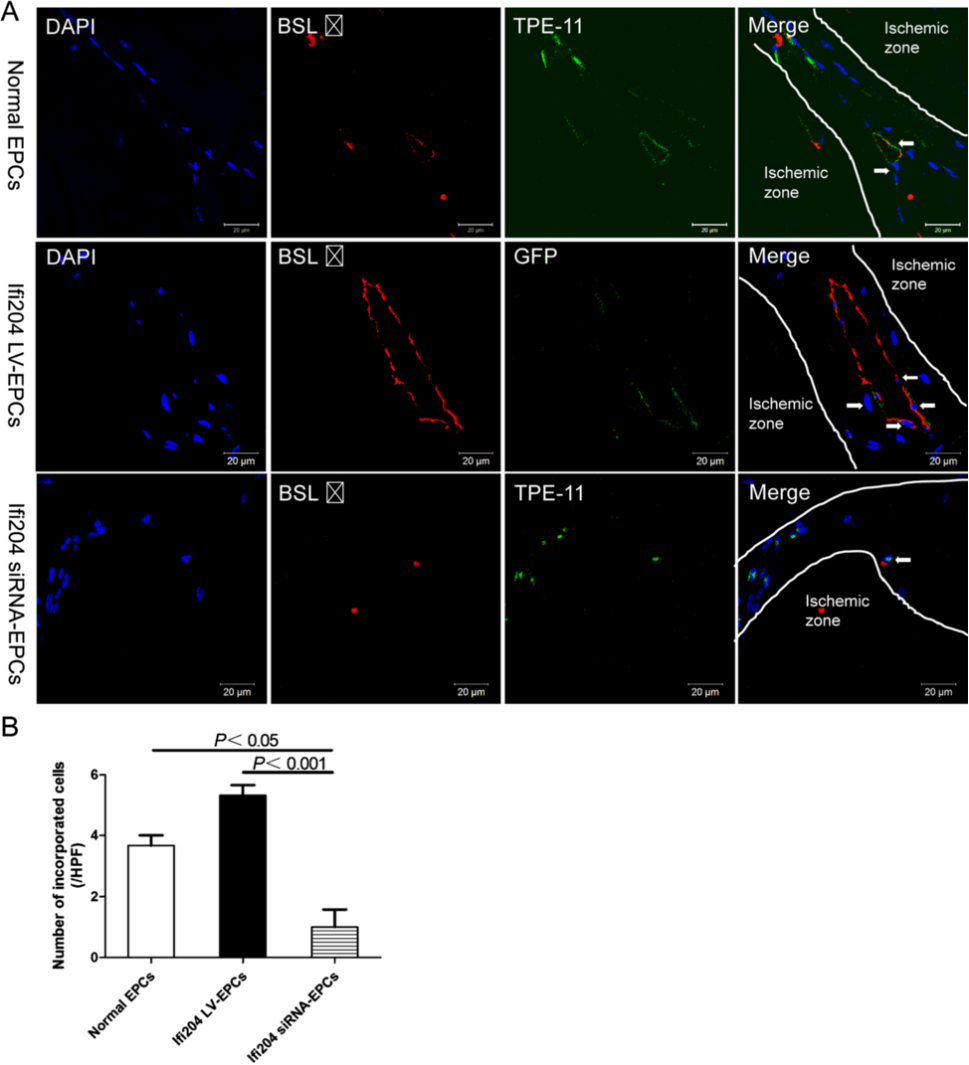
Detection of cell incorporation into neovessels in ischemic hindlimb. Nonmodified EPCs and Ifi204 siRNA-transfected EPCs were first labeled with TPE-11, and then were intramuscularly injected together with Ifi204 lentivirus-transduced EPCs immediately after hindlimb ischemia. Muscle samples were harvested following BSL I systemic perfusion 14 days after surgery. **a** Colabeling with BSL I (*red*) and TPE-11 (*green*) appears (*orange*, marked by *white arrows*) in the section of ischemic calf muscle. *Bar*, 20 μm. **b** Numbers of incorporated cells into vessels were counted and averaged (*n* = 4). *P* < 0.05, normal EPCs vs Ifi204 siRNA-EPCs; *P* < 0.001, Ifi204 LV-EPCs vs Ifi204 siRNA EPCs. *EPC* endothelial progenitor cell

Revascularization of the ischemic hindlimb during the 14 postoperative days was additionally evaluated by immunohistochemistry. The capillaries in the ischemic area are visualized by immunofluorescent staining with an antibody to isolectin B4, which stains endothelial cells, and counted in representative high-power fields (HPF) (Fig. 7a). The mice transplanted with Ifi204 overexpressing EPCs and normal EPCs exhibited sufficient growth of neovessels, while the mice transplanted with Ifi204 siRNA-transfected EPCs exhibited less neoangiogenesis (*P* < 0.001, Ifi204 siRNA-EPCs: 13 ± 1.7/HPF vs normal EPCs: 24 ± 1.7/HPF and Ifi204 LV-EPCs: 27 ± 2.5/HPF) (Fig. 7b). A newly-synthesized nanofluorogen, a bolaamphiphile with a tetraphenylethene (TPE) unit attached and two pyridinium salt terminated alkyl groups (TPE-11), was used to track the implanted cells. The fluorescent spots can be clearly seen in the cytoplasmic area of EPCs at the excitation wavelength of 405 nm, and the intensity in the nuclei region is relatively weak (Fig. 8a). In the sections of ischemic hindlimb, we could also detect the cells with TPE-11 labeling (Fig. 8b). EPCs with overexpression of Ifi204 incorporated more into vessel formation, but downexpression of Ifi204 inhibited this stimulatory effect under the control level (Fig. 9a, b). These data indicated that the stimulation of angiogenesis induced by EPCs in ischemic hindlimb was related with the expression level of Ifi204.

## Discussion

Interferon-inducible protein 204 (Ifi204) was shown to be an important mediator for the differentiation of osteoblasts [24–26], skeletal muscle myoblasts [16, 27], and cardiomyocytes [28, 29], previously. Ifi204 has been documented to overcame the inhibition of transcription factors such as Gata4, Nkx2.5, MyoD, and Cdfa1 by sequestering inhibition of differentiation (Id) proteins, thus promoting differentiation of cardiomyocytes and osteoblasts [17]. However, the role of Ifi204 in differentiation of EPCs remains unknown. Very recently, our group reported that Ifi204 is downregulated in the bone marrow cells of diabetes mellitus type 2 mice [18]. Because EPCs in diabetes mellitus have been reported to be impaired in regenerative potency, we thus proposed that Ifi204 could modulate the differentiation and regenerative abilities of EPCs. Our study indicates that downregulation of Ifi204 in EPCs significantly inhibited their endothelial differentiation and the angiogenic potencies in vitro and in vivo, while upregulation of Ifi204 had the opposite effects. To our knowledge, this is the first study into the regulatory role of Ifi204 on the differentiation and functions of EPCs.

As evidenced by RT-qPCR, immunocytochemistry, and FACS, upregulation of Ifi204 in EPCs promoted endothelial differentiation of the cells and vice versa. One of the paracrine factors of VEGF was also secreted more in EPCs infected with Ifi204 lentivirus compared with nonmodified and Ifi204 siRNA transfected cells, whereas cell proliferation was not affected by Ifi204 expression levels (Additional file 1: Figure S1). The mechanisms for how Ifi204 affects the differentiation and paracrine signature of EPCs were unclear and worthy of further investigation. EPCs by themselves do not generate tubes in vitro in Matrigel [30], so researchers utilized EPCs and HUVECs to perform the Matrigel assay [19–21]. Vascular tube formation in Matrigel is thought to recapitulate in-vivo vasculogenesis and angiogenesis [30]. The more angiogenic potencies that EPCs have, the more easily the cells could incorporate into tubular structures of HUVECs. An in-vitro incorporation study also demonstrated that the Ifi204 lentivirus-transduced EPCs could incorporate more into the tubes of HUVECs. To verify the effect of Ifi204, modified EPCs were transplanted into ischemic hindlimb. In mice with hindlimb ischemia, downregulation of Ifi204 in EPCs impaired endothelial differentiation, with a corresponding reduction in neovascularization and hindlimb blood reperfusion by postoperative day 14. The present study provided evidence that Ifi204 is required for endothelial differentiation of EPCs and the angiogenesis ability of EPCs was modulated by the expression of Ifi204. These findings are consistent with previous reports from other researchers that Ifi204 has participated in the regulation of cell differentiation. Further studies elucidating the downstream signaling molecules of Ifi204 in the modulation of EPC differentiation are needed.

EPCs can form colony-forming units (CFU) in methylcellulose supplied with growth factors. The colony-forming assay determines the stemness of EPCs, and colonies are classified into two types according to their morphology: primitive (small cell) colonies and definitive (large cell) colonies [19, 31]. The primitive CFU is a predominantly proliferative population of cells and the definitive CFU is a predominantly vasculogenic population with greater adhesion, migration, differentiation, and tabularization potential. EPCs downexpressing Ifi204 formed less large cell colonies than those overexpressing Ifi204, which indicates that Ifi204 inhibition may block the stemness and vasculogenic potency of EPCs and supports all of our findings.

TPE-11 is a nanofluorogen newly synthesized by our group. We have already used it in the labeling of Hela cells, and the biocompatibility of the nanofluorogens formed by TPE-11 was quite good in cells [23]. TPE-11 can be transported and passaged to the progeny cells by microtubules (unpublished observations). We also found that TPE-11 can be retained stably in the embryoid of the embryonic stem cells (unpublished observations: Shixin Zhou (S. Z.), Yijun Xia (Y. X.), Yinan Liu (Y. L.), Qihua He (Q. H.) and Bo Song (B. S.). 2016.). As far as we are aware, this work represents the first experimental demonstration of in-vivo cell labeling using this emerging luminescent system.

## Conclusions

This study makes two contributions to EPC-mediated therapy of hindlimb ischemia. First, Ifi204 is required for EPC differentiation and neovascularization in vitro and in vivo. Second, EPCs labeled with TPE-11 could be detected in the ischemic hindlimb. The regulatory roles of Ifi204 in EPC differentiation need to be elucidated further and may benefit the therapeutic strategies for ischemic vascular diseases.

## Abbreviations

BSL I, Griffonia (Bandeiraea) Simplicifolia Lectin I; CFU, colony-forming unit; EPC, endothelial progenitor cell; FACS, fluorescence-activated cell sorting; Ifi204 LV-EPCs, EPCs transduced with Ifi204 lentivirus; Ifi204 siRNA-EPCs, EPCs transfected with Ifi204 siRNA; Ifi204, interferon-inducible protein 204; LDPI, laser Doppler perfusion imaging; RT-qPCR, reverse transcription quantitative PCR; TPE, tetraphenylethene; HUVEC, human umbilical vein endothelial cell; TPE-11, a bolaamphiphile with a tetraphenylethene unit attached and two pyridinium salt terminated alkyl groups; HPF, high-power field

## Additional file

Additional file 1: Figure S1. Showing the detection of cell proliferation by CCK-8 assay. **A** Cells were transfected with Ifi204 siRNA and control siRNA. OD values were read after 2 hours of incubation with CCK-8 solution. **B** Cells were infected with Ifi204 LV5 and control LV5. OD values were read after 2 hours of incubation with CCK-8 solution. (JPG 41 kb)
