# Supplementary figures and images for: Regulatory roles of interferon-inducible protein 204 on differentiation and vasculogenic activity of endothelial progenitor cells
Source: Stem Cell Res Ther. 2016 Aug 11;7:111. doi: 10.1186/s13287-016-0365-5 (PMC4981987; doi:10.1186/s13287-016-0365-5)

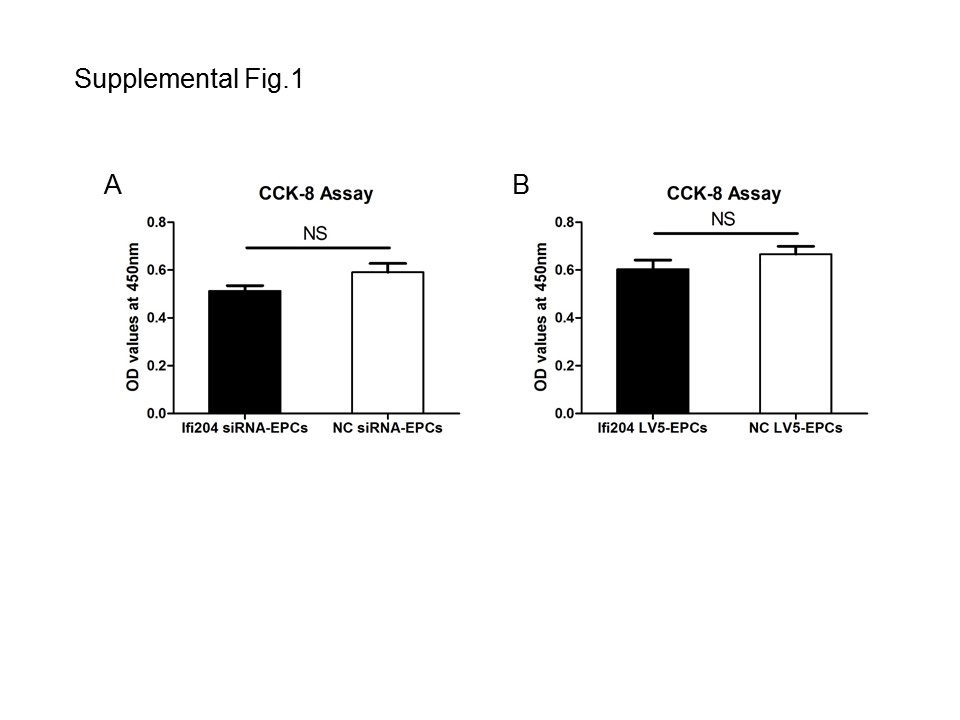

Supplement: Additional file 1: Figure S1. — Showing the detection of cell proliferation by CCK-8 assay. A Cells were transfected with Ifi204 siRNA and control siRNA. OD values were read after 2 hours of incubation with CCK-8 solution. B Cells were infected with Ifi204 LV5 and control LV5. OD values were read after 2 hours of incubation with CCK-8 solution. (JPG 41 kb) [file 13287_2016_365_MOESM1_ESM.jpg]
